# Inability to Perform Retrograde Ureteroscopic Stone Treatment: A Postoperative Anatomical Change After Fleur-de-Lis Abdominoplasty

**DOI:** 10.1155/criu/4992363

**Published:** 2025-05-21

**Authors:** Parker L. Heger, Thomas F. Rashid, Lucas B. Vergamini, Bristol B. Whiles, Aaron Tverye, Wilson R. Molina

**Affiliations:** ^1^Department of Surgery, University of North Dakota School of Medicine and Health Sciences, Grand Forks, North Dakota, USA; ^2^Department of Urology, University of Kansas Medical Center, Kansas City, Kansas, USA

## Abstract

Abdominoplasty is a frequently performed elective procedure, often indicated for patients after substantial weight loss resulting in significant redundant skin. Closing techniques and the lifting of the mons pubis during abdominoplasty have been proposed to alleviate symptoms of stress urinary incontinence by elevating and supporting the urethra. Despite these observations, the impact of abdominoplasty on pelvic anatomy and subsequent endoscopic procedures remains underexplored and underreported. We present a case where cystoscope passage as part of endoscopic laser lithotripsy for nephrolithiasis was impeded by altered anatomy in a patient with a history of Fleur-de-Lis abdominoplasty.

## 1. Introduction

Abdominoplasty is a prevalent elective surgical procedure commonly performed following substantial weight loss to reduce excess skin and remaining adipose tissue. While there is evidence suggesting that abdominoplasty may influence pelvic anatomy and urinary symptoms, the implications for subsequent endoscopic interventions on the urinary tract for other conditions are not well documented. This case highlights the challenges encountered during ureteroscopy with laser lithotripsy in a patient with prior Fleur-de-Lis abdominoplasty while emphasizing the need for further investigation into these anatomical changes after prior surgery.

## 2. Case Presentation

A 52-year-old male with a history of Roux-en-Y gastric bypass, Fleur-de-Lis abdominoplasty, pubic soft tissue resection, bilateral brachioplasty, bilateral simple mastectomy, bilateral thigh lift, and nephrolithiasis presented with colicky abdominal pain, nausea, and vomiting. Noncontrast CT revealed a 0.8-cm calculus at the right ureteropelvic junction (UPJ). Initial management with trial of passage including medical expulsive therapy with tamsulosin failed, leading to a planned outpatient cystoscopy with right ureteroscopy and laser lithotripsy for definitive stone management.

**Figure 1:**
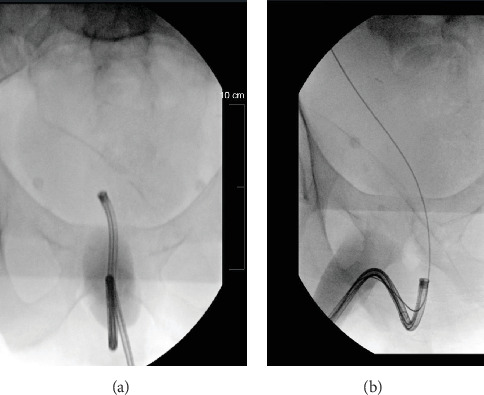
(a) Anterior to posterior and (b) lateral x-ray views of flexible cystoscope course to the bladder including safety wire tortuosity with wire advanced into the right upper tract.

**Figure 2:**
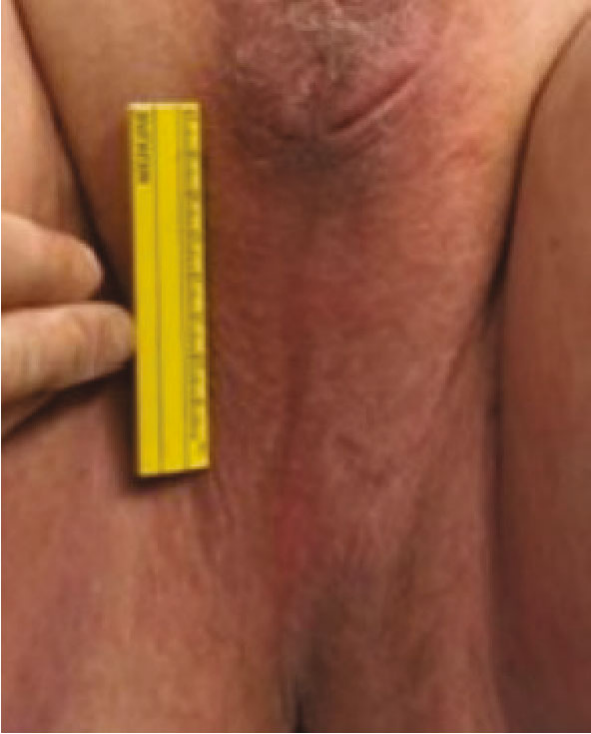
Perineal length with ruler showing abnormally long distance from scrotum to the anus.

**Figure 3:**
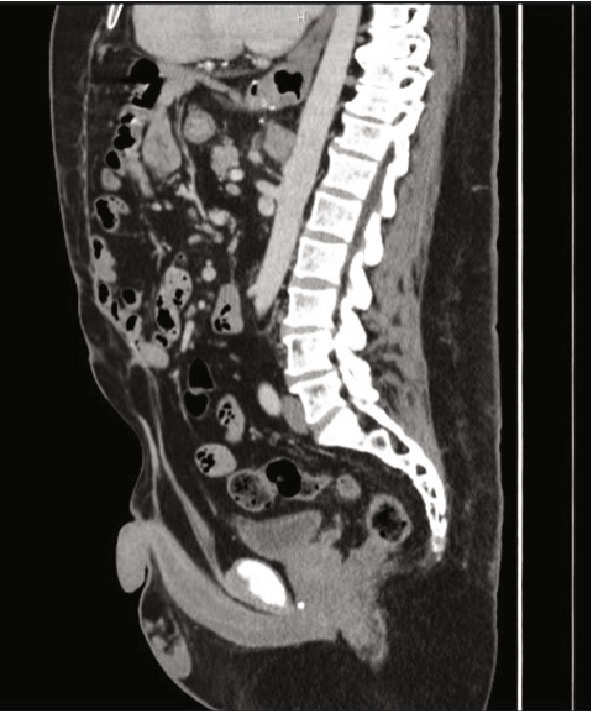
Sagittal CT demonstrating high bladder neck and abnormal penoscrotal anatomy.

The patient was placed in the dorsal lithotomy position. During the procedure, a 22-Fr rigid cystoscope with a 30° lens was inserted into the urethral meatus and advanced toward the bladder. However, the scope could not be advanced proximal to the verumontanum due to a high upward angle. A flexible cystoscope was employed to navigate the long penile and proximal urethra to access the bladder, where the right ureteral orifice was identified and appeared tight. A sensor wire was placed into the right ureteral orifice and advanced to the renal pelvis; however, when advancing a flexible ureteroscope or an 8–10-Fr ureteral dilator, severe coiling of the wire in the proximal urethra was observed (Figures 1 and 2), likely attributable to the patient's long perineum resulting from the prior abdominoplasty. Further review of the patient's previous CT scan also demonstrated a high bladder neck and tortuous urethra (Figure 3). Attempts to mitigate the coiling with a Pollock catheter and a superstiff guidewire were unsuccessful. Maneuvers to alter the urethral anatomy to facilitate navigation, such as pulling the penis, were not effective. Ultimately, due to the inability to advance a flexible ureteroscope or access sheath past the proximal urethra due to a high bladder neck, a 6‐Fr × 30-cm ureteral stent was placed with the plan to return to the operating room for right ureteroscopy with laser lithotripsy with possible right percutaneous nephrolithotomy in the future if retrograde access was not possible after passive ureteral dilation with a ureteral stent.

**Figure 4:**
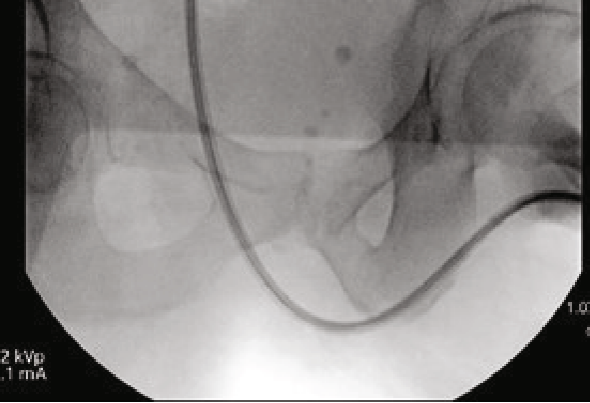
Lateral view of ureteral access sheath during the second case demonstrating significantly less urethral tortuosity due to altered patient positioning.

The patient returned to the operating room approximately 4 weeks later. He was positioned in the supine split-leg position in anticipation of a possible right percutaneous nephrolithotomy. However, a wire was passed through the existing ureteral stent, and there was significantly less tortuosity of the urethra due to the patient's positioning. There was no coiling of the guidewire on retrograde urethrogram (Figure 4). An 11–1‐Fr × 36-cm access sheath was carefully passed without resistance. Given the ability to achieve retrograde access, we proceed with right ureteroscopy and laser lithotripsy. The case was uncomplicated, and a 6‐Fr × 30-cm ureteral stent was placed at the conclusion of the case. Patient was found to have no residual stone disease on follow-up.

## 3. Discussion

According to the American Society of Plastic Surgeons, 170,110 abdominoplasties were performed in the United States in 2023, making it the third most common cosmetic procedure [1]. The Fleur-de-Lis variant includes a vertical incision in addition to the horizontal one and is typically reserved for patients with significant weight loss. It is considered a safe alternative with similar complication rates to the normal technique [2].

While abdominoplasty is commonly associated with improved stress urinary incontinence (SUI) symptoms, the impact on pelvic anatomy and its potential influence on future endoscopic procedures or difficulties are not well documented. A study done by Carruthers et al. reported a 60% reduction in SUI symptoms after abdominoplasty [3]. Another study done by Taylor et al. found very similar results and noted that patients found symptom relief regardless of the abdominoplasty technique used [4]. The precise mechanism remains unclear but may have to do with closing techniques that support the urethra or elevating the mons pubis to increase the stability of the urethra. One study found that the addition of a mons pubis lift procedure during abdominoplasty helped improve SUI symptoms [5]. While literature demonstrating the impact of abdominoplasty on subsequent endoscopic urologic procedures is scarce, the impact of abdominoplasty on SUI documented in the literature can likely be attributed to anatomical alterations to the urethra. To our knowledge, this is the first case describing difficulties gaining retrograde access during an endoscopic urologic procedure.

The inability to achieve retrograde access during the first case was multifactorial. The patient had a narrow right ureteral orifice that required dilation for retrograde access. However, the patient's tortuous urethra and high bladder neck led to coiling of the guidewire, which prevented the insertion of a ureteral dilator during the first procedure, which concluded with the placing of a ureteral stent. During the second look, changing the patient's position (supine split-leg instead of dorsal lithotomy) led to a less tortuous urethra. Passive dilation of the ureter with an indwelling ureteral stent also allowed for easier retrograde access. In this scenario, the combination of optimized patient positioning and ureteral dilation made retrograde access with ureteroscopy possible.

## 4. Conclusion

Abdominoplasty, particularly the Fleur-de-Lis variant, can significantly alter pelvic anatomy, affecting the approach success of future endoscopic procedures. This case illustrates a unique complication where anatomical changes from prior abdominoplasty obstructed retrograde endoscopic access for kidney stone treatment. Given the increasing prevalence of abdominoplasty, awareness of this potential postprocedural difficulty due to anatomical changes is crucial for urologists to optimize patient management. Careful review of urethral anatomy on cross-sectional imaging as well as optimized patient positioning can be helpful to mitigate these procedural challenges. Furthermore, conservative management with prestenting can make retrograde access easier due to passive ureteral dilation.

## Data Availability

Data sharing is not applicable to this article as no new data were created or analyzed.
